# Silk Fibroin as an Efficient Biomaterial for Drug Delivery, Gene Therapy, and Wound Healing

**DOI:** 10.3390/ijms232214421

**Published:** 2022-11-20

**Authors:** Shahid Ud Din Wani, Mohammed Iqbal Zargar, Mubashir Hussain Masoodi, Sultan Alshehri, Prawez Alam, Mohammed M. Ghoneim, Areej Alshlowi, H. G. Shivakumar, Mohammad Ali, Faiyaz Shakeel

**Affiliations:** 1 Department of Pharmaceutical Sciences, School of Applied Science and Technology, University of Kashmir, Jammu and Kashmir, Srinagar 190006, India; 2 Department of Pharmaceutical Sciences, College of Pharmacy, AlMaarefa University, Ad Diriyah 13713, Saudi Arabia; 3 Department of Pharmacognosy, College of Pharmacy, Prince Sattam Bin Abdulaziz University, Al-Kharj 11942, Saudi Arabia; 4 Department of Pharmacy Practice, College of Pharmacy, AlMaarefa University, Ad Diriyah 13713, Saudi Arabia; 5 Department of Pharmaceutics, College of Pharmacy, JSS Academy of Technical Education, Noida 201301, India; 6 Department of Pharmacy Practice, East Point College of Pharmacy, Bangalore 560049, India; 7 Department of Pharmaceutics, College of Pharmacy, King Saud University, Riyadh 11451, Saudi Arabia

**Keywords:** silk fibroin, biopolymers, biomaterials, drug delivery applications, wound healing, gene therapy

## Abstract

Silk fibroin (SF), an organic material obtained from the cocoons of a silkworm *Bombyx mori*, is used in several applications and has a proven track record in biomedicine owing to its superior compatibility with the human body, superb mechanical characteristics, and its controllable propensity to decay. Due to its robust biocompatibility, less immunogenic, non-toxic, non-carcinogenic, and biodegradable properties, it has been widely used in biological and biomedical fields, including wound healing. The key strategies for building diverse SF-based drug delivery systems are discussed in this review, as well as the most recent ways for developing functionalized SF for controlled or redirected medicines, gene therapy, and wound healing. Understanding the features of SF and the various ways to manipulate its physicochemical and mechanical properties enables the development of more effective drug delivery devices. Drugs are encapsulated in SF-based drug delivery systems to extend their shelf life and control their release, allowing them to travel further across the bloodstream and thus extend their range of operation. Furthermore, due to their tunable properties, SF-based drug delivery systems open up new possibilities for drug delivery, gene therapy, and wound healing.

## 1. Introduction

Polymeric formulations have developed as a modern alternative to conventional formulations of maintaining a supply of active pharmaceutical ingredients (APIs), optimizing their physicochemical properties, maximizing their effectiveness, and resolving many crucial issues in drug delivery, such as unique intracellular targeting transportation and biocompatibility in the process of optimizing therapeutic efficacy and patient quality of life [1,2,3,4]. In the case of incredibly harmful medications such as anti-cancer drugs, an optimal drug delivery mechanism would soothe the loaded drug, allowing it to regulate its kinetic releases, and reduce its unfavorable effects through tissue-specific targeting. For decades, silk has been recognized as an important natural material for fabric production; but in recent years, it piqued interest as a possible biopolymer for biomedical and pharmaceutical applications [5,6,7].

Several attempts have been made in recent years to develop and build different forms of nano-scale drug carriers to encapsulate corrective particles and thus unleash the drug under controlled conditions [8,9,10]. The development, as well as the formulation of new systems of drug delivery, is an exciting and rapidly growing area. The ease of access to appropriate material, the material’s complete safety to the host, and the material’s important physic-chemical and biomedical characteristics, as well as the ability to a breakdown in biological environments, are all important requirements for an outstanding drug delivery method. For drug distribution, a variety of processing materials, such as synthetic and natural polymers, have been used. So many synthesized polymers that are widely used are made with one or two monomers and have a low deterioration speed. Biodegradation in vivo can be predicted by factors such as the mass of molecules or the composition of monomers. In contrast to synthetic fibers, organic polymers have outstanding biocompatibilities as well. Numerous types of nano-particles made from artificial or organic polymers have been confirmed to be in initial clinical trials for the diagnosis of diabetes, tumor, and other sicknesses. Silk fibroin (SF) is a naturally occurring protein derived mostly from cocoons of the silkworm, *Bombyx mori*, and successfully utilized in biomedicine because of its supreme biocompatibility, mechanical behavior, and tunable biodegradability [11,12,13,14,15,16,17,18]. The rate of SF degradation is being managed by adjusting its molecular mass, crystalline size, or cross-linking [19,20,21].

A growing number of systems focused on SF are being used to store and distribute medicines in recent years [22,23,24,25,26,27,28]. The efficiency of drug distribution and loading in such silk drug systems is determined by its hydrophobic nature and charge, which point to several drug-release contours. Furthermore, diverse forms of delivery mechanisms such as films, hydrogels, microspheres, nanoparticles, and scaffolds may be made from silk fibroin (SF) solutions using various techniques [29]. Silk I, Silk II, and Silk III are the three structural forms of SF. Silk I is a hydrophilic protein that contains a great proportion of domains of α-helix as well as random coils [30]. Silk II, on the other hand, has a structure of β-sheet mostly that is extra stable and not water soluble, whereas Silk III dominates only at the water–air interface [31]. As a result, natural and artificial polymers can be used to develop and fabricate new therapeutics. Li and his colleagues used electrospray to build cisplatin-encapsulated SF nanostructures without utilizing organic solvents [32]. The drug particles were inserted into nanomaterials using a metal-polymer arrangement of bond sharing, as well as the drug could be released at slow speed and sustainably for fifteen days and more, according to the in vitro release studies.

**Figure 1:**
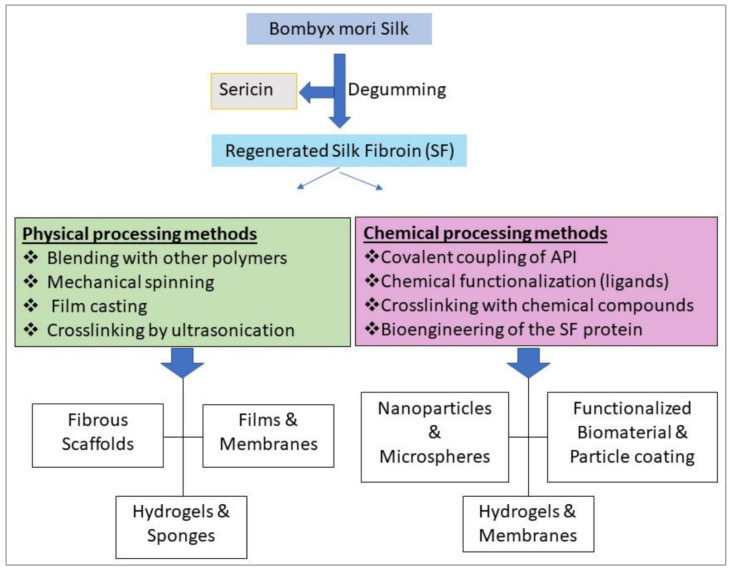
For pharmaceutical and biomedical implementations, a versatile range of functional arrangements and chemical treatment for manufacturing a variety of silk fibroin (SF) formats are available. Image reproduced from reference [39] which was published under a CC BY license.

The coupling of chemicals and the genomic alteration of silk-protein through chaining the amino acid sequence or inserting a segment to achieve a certain feature are the two major techniques for functionalizing silk proteins [33]. A huge number of antitumor dosage forms are formulated for parenteral delivery, resulting in close interaction with blood products. The therapeutic agents used in these preparations must not cause hematological poisoning or immune reactions [3], as a result, biocompatible polymers must be used in the preparation. Besides that, developing delivery mechanisms for therapeutic products including vaccines and antibodies necessitates ensuring both their physical integrity and biological activity, which is especially important for controlled-release mechanisms [34]. Utilizing non-modified or engineered SF protein production and formulating techniques, a broad variety of therapeutic agents of varying sizes and morphologies can be prepared (Figure 1). SF transports that have not been changed have been used to carry antitumor drugs such as doxorubicin [35], paclitaxel [36], curcumin [9,37], and cisplatin [38]. In the present review, the key techniques for developing various SF-based drug delivery systems, as well as the most recent methods for developing functionalized SF for managed or redirected drugs, gene therapy, and wound healing, are addressed.

## 2. Processing of SF Biomaterials or Silk Cocoon Processing—Generating Silk for Biomedical Applications

Degumming of the silk cocoon of *Bombyx mori* to extract sericin is one of the most important steps in producing silk appropriate for biomedical application. Enzymatic approaches (digestive process sericin not silk) or production of chemicals may be used to isolate sericin (example- treatment of alkaline). The latter process is generally utilized, and sodium bicarbonate is used to boil silk thread over 20–60 min [40].

**Figure 2:**
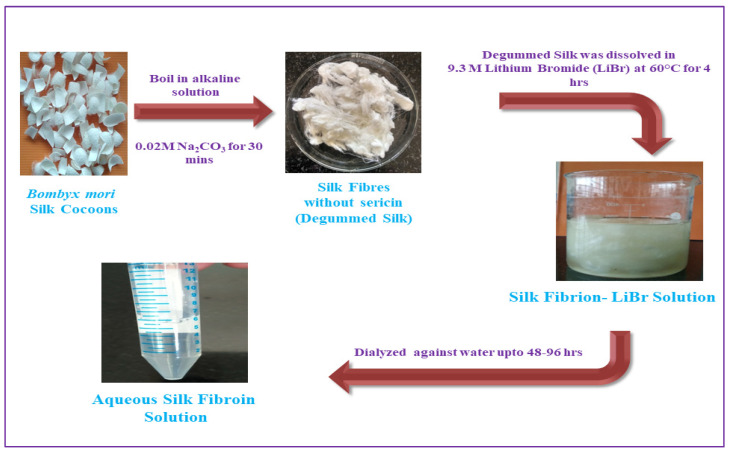
Processing of SF.

Degumming periods as limited as five minutes are also adequate to eliminate sericin while mitigating silk destruction, which is typically caused by the disulfide’s cleavage connection between the heavy as well as light chains and separation of a sequence of amorphous silk throughout the heavy chain, resulting in polydispersed silk [41]. By dissolving the degummed cotton thread in such a chaotropic agent’s large concentration (for say, lithium bromide 9.3 M) over 60 °C for numerous hours, the silk framework of higher level can be completely reverse engineered. The resultant regenerated silk fibroin liquid is therefore dialyzed with water vigorously to create a silk aqueous solution which is steady at room temperature over half a month as well as at 4 °C over months (Figure 2) [40]. The whole reverse modified silk fibroin formulation has a lower solution conformation than native silk feedstock [42] and rheological properties are altered [43]. Novel silk materials, such as scaffolds, films, fibers, and (self-assembling) silk hydrogels, as well as (nano) fragments and (nano) abrasives, are frequently created using a reverse-optimized aqueous-regenerated silk fibroin solution within ambient circumstances. This gentle processing environment is perfect for maintaining biological function.

## 3. Properties of SF

SF has a rare balance of mechanical and biological attributes, due to its natural polymer-like characteristics [44,45]. Silk is often associated with a soft texture in the garment industry, but its tensile capacity and modulus make this one of the most durable natural biomaterials [46]. This property is crucial for polymers used in bone tissue reconstruction since the polymer’s mechanical efficiency is critical in such implementations [47]. Under the thermal stress (less than that of 250 °C), SF is highly stable. Lu et al. reported that differential scanning calorimetry results revealed that Silk I crystals had stable thermal properties up to 250 °C, without crystallization above the Tg, but degraded at lower temperatures than Silk II structure [48].

### 3.1. Physiochemical Characteristics of SF

SF has a one-of-a-kind blend of mechanical as well as biological characteristics, as well as unusual characteristics of both artificial and natural polymers [49,50]. While silk’s tensile strength and modulus make it one of nature’s most durable biomaterials, it is also synonymous with a soft texture in the garment industry [51]. This ability is essential for polymers used in bone tissue regeneration because mechanical performance is important in such implementations [52,53].

### 3.2. Mechanical Properties

For pharmaceutical and biomedical uses, mechanical toughness is an essential characteristic of SF-based formulations. For example, the strength of the SF product use for tissue engineering must equal that of the specific tissue. The hardness of the SF polymer will also influence its durability and degradability [54]. Numerous polymers used within drug distribution systems such as PLGA and collagen, are not solid enough mechanically. Cross-linking is a common technique for the mechanical properties of biopolymers such as collagen. The cross-linking interaction, on the other hand, may have unfavorable effects, such as immunogenicity and mitochondrial toxicity [55]. SF has a solid β-sheet construction that allows it to have outstanding mechanical characteristics without having any severe crosslinking procedures. SF can be transformed into a variety of formats, comprising liquid, hydrogels, and scaffolds depending on the material of the β-sheet [5]. Young’s modulus is commonly used to measure mechanical strength using nanoindentation strategies [56]. Because of its strong tensile strength and compressive force resistance, SF is an excellent material for drug delivery and tissue engineering [57]. Furthermore, extracting sericin during the degumming process increases the tensile strength of SF by 50% [58], rendering it more durable during physical pharmaceutical manufacturing.

### 3.3. Stability

Amongst the very important considerations in the manufacture of pharmaceutical formulations is the stability of polymeric products. Biopolymers are favored over synthetic alternatives for therapeutic uses because of their biocompatibility and biodegradability but they should also follow such durability requirements to be accepted for use in the pharmaceutical companies. Accumulation or gelation through extended preservation is among the most common issues with pure SF solutions. SF comes in two varieties: insoluble (high β-sheet content) and soluble (high α-helix and random coil content). Either type should be utilized and preserved, based on the medication preparation. As soluble SF is processed in extremely humid circumstances, it undergoes a transition from α-helix and random coil to β-film, that can result in gel formation and a reduction in the SF solution’s stability [59,60]. In comparison to other proteins, SF has superior heat durability. The temperature at which glass transitions (Tg), which is influenced by the β-sheet content of silk fiber, is the best measure of protein heat stability. SF film’s Tg is about 175 degrees Celsius, and the protein stays steady up to 250 degrees Celsius, which is ideal for product manufacturing. The Tg of frozen SF solution, on the other hand, is about −34~−20 °C [61], which is also advantageous in medication manufacturing at low temperatures.

### 3.4. Degradability and Biocompatibility

The FDA has formally approved silk fiber as a biocompatible substance for the application of a variety of nanotechnological instruments [62]. Silk’s biocompatibility has been thoroughly researched over the last two decades. In contrast to other commonly used biological polymers that degrade within the industry of pharmaceuticals, including collagen, poly(lactic-co-glycolic acid) (PLGA), and polylactide (PLA), the vast majority of researches have indicated a less immunogenic response and outstanding biocompatibility [63,64,65]. Mesenchymal stem cells, fibroblasts, hepatocytes, endothelial cells, and osteoblasts were shown to be highly compatible with SF configurations in cytocompatibility studies (MSCs) [66,67]. Methanol and hexafluoroisopropanol (HFIP) are used as organic solvents in SF manufacturing to cross-link SF by causing structural modification (α-helix to β-sheet), that linked to the SF formulations’ inflammatory ability [68]. To stop these inflammatory reactions, however, slightly different manufacturing methods have been used to avoid the use of organic solvents [69].

The capacity of biological materials to degrade is an essential attribute. Even though biodegradability is a key benefit of SF in medicinal implementations it also makes pure silk fiber particles susceptible to enzymes that break down proteins. The pace at which SF deteriorates can also be managed by adjusting the molecular mass, crystalline nature, morphological characteristics, or cross–linking [70]; however, cross-linking and the level of the crystalline phase is not the only option to consider to prevent SF from deteriorating. When SF sheets were subjected to collagenase IA in an in vitro enzymatic destruction test, the crystalline shape of the sheets changed from Silk II to Silk I. While the protease XIV enzyme was used, however, the bulk of the SF films were converted to Silk I, resulting in stronger crystallinity. Although both enzymes took 15 days to degrade, the degradation rate of protease XIV was slightly lower than that of collagenase IA [71]. Another research showed that SF deterioration induces a predictable loss of mechanical integrity [72].

## 4. SF-Based Biomaterial for Drug Delivery Systems

Many therapeutic applications need active pharmaceutical ingredients (APIs) to be delivered in continuous and managed release modes. The scale, composition, and other characteristics of the particles are determined by the form of the distribution device and the administration path. Furthermore, in such distribution systems, using polymers that are biocompatible and mechanically robust with moderate conditions of fabrication and manufacturing is beneficial for maintaining the loaded API’s bioactivity. As previously mentioned, SF fits both of these conditions, making it a successful candidate for drug delivery [5,73]. Nanofibers, films, lyophilized sponges, SF-coated polymeric particles, hydrogels, and micro-and nanoparticles are only some of the SF-based delivery systems of drugs that have been created. Many of the most commonly researched drug delivery of SF-based mechanisms are discussed in the following section.

### 4.1. Hydrogels

The SF aqueous phase was used in a variety of ways to make hydrogels. Physiochemical or chemical methods involving organic polymers or artificial reagents may initiate the conversion from solution to gel [74]. Water evaporation or osmotic stress, shearing (spinning), electric field, and warming are some of the physicochemical operations. The gel shape is stabilized by the stability in terms of thermodynamics β-sheets, which create a stable gel-type under physiological circumstances before enzymes or oxidative processes destroy it extensively [75]. Gel scaffolds containing curcumin designed by electro-gelation were used in a recent analysis for wound healing. Not only did the formulated gel formulation boosts protein adsorption and curcumin release, but it also boosted the bacterial inhibitory effect six-fold against S. aureus [76]. Protein adsorption on substrates has been known for a long time to have a vital factor in cell development and regeneration; SF gel scaffolds may help in tissue repair by encouraging cell proliferation.

### 4.2. Silk Films

As a biomaterial, SF has massive potential in medication formulations, tissue engineering, and in the processing of films from SF [77]. Casting an aqueous SF solution is a simple way to make SF films [78]. Certain SF film preparing strategies have been published such as a vertical deposition [79], spin coating [80], and spin-assisted layer-by-layer assembly [81]. Rather than binding independently to the hydrogel base, fibroblasts are aggregated on the rigid surface. Terada et al. [71] looked at how spin-coated SF films acted when subjected to varying ethanol concentrations. A jelly-like hydrogel coating was created with alcohol concentrations of less than 80%, while a solid film surface was created with alcohol concentrations of more than 90%. The binding of fibroblast cells to SF films was influenced by this shift in morphology [80].

### 4.3. Silk Particles

Nanoparticle drug delivery mechanisms have been researched the most, particularly for chemotherapeutic agents, among systems based on SF that are utilized for enclosing APIs and completing drug distribution modulation. The SF nanoparticles of lysosomotropic engineered by Seib et al. [23] for pH-dependent activation of the antitumor agent’s doxorubicin in sequence to counteract drug resistance are one example of such structures. SF nanomaterials are primarily used to deliver the primed drug to the target site in a controlled manner. SF nanoparticles can be made using a variety of processes, such as polyvinyl alcohol (PVA) blends, that can be designed to make silk fiber spheres of new shapes and sizes [81] (Table 1). The charge and lipophilicity of such systems are deciding factors for drug delivery and encapsulation quality. Different drug release profiles result from changing these conditions [82]. Furthermore, the inclusion of PVA greatly increases the morphology of the SF particles [83]. The salting-out process is one of the most common ways to make SF particles. Lammel et al., for example, used a salting-out agent potassium phosphate to create particles of SF with manageable sizes from 500 nm to 2 µm [84]. Tian et al. used the salting-out process to make SF nanoparticles, which were then filled with Fe_3_O_4_ magnetic nanomaterials and doxorubicin and guided to the tissue of interest utilizing an additional magnetic field to achieve tissue-specific guided delivery [85]. It was discovered that modifying the concentration of Fe_3_O_4_ in the formulation would affect the doxorubicin entrapment performance [85]. Moreover, current research on microfluidics used a desolvation approach to generate tinier SF spheres (150–300 nm) using a microfluidic setup (nano-assembler) [86]. The characteristics of SF nanoparticles were discovered to be influenced by two major factors: rate of flow and percentage of flow rate [87]. The use of a microfluidic device allowed for the development of SF nanomaterials with desired sizes for drug delivery that was fast, repeatable, and monitored. Monitoring the particle size and zeta potential allows for fine tuning of drug delivery.

## 5. SF-Based Biomaterials for Biomedical Applications

Minute particles of medicine [3], biological API drugs [91], and genes [91] have all been distributed via silk. Various silk production methods have been used to create numerous compositions for each category of therapeutic agents [92]. Stabilizing the formulated API and adjusting its circulation period to produce the desired therapeutic result are important key requirements of delivery systems based on SF. Furthermore, engineered formulations are often tailored for a specific use of drug distribution, such as preloaded drug stabilization, monitoring drug discharge, or optimizing cell adhesion [93]. The following section will include a description of SF implementations in the delivery of drugs and genes, with the review given in (Table 2 and Table 3).

### 5.1. SF Helps to Keep Drugs Stable

The primary aim of integrating active compounds such as minute particles or peptides into SF-based reservoirs is to stabilize them through a range of processes such as covalent interaction, adsorption, and/or enslavement [101]. Sustained drug release is difficult to perform without a safe relationship between the SF-based reservoir and drug to hold the drug active. Away from minute cases, such as the majority of stabilization and growth factors strategies focus on uniformly spreading the substance inside the SF-matrix/particles [26]. Temperature [102], humidity [103], and pH [104] all affect the stability of SF-based biomaterials. As a result, they have been extensively researched for improving the durability of other compounds, such as the encapsulation of antibiotics such as erythromycin, which has poor water stability.

### 5.2. Drug Delivery

Several investigators have concentrated on silk as a model or developing novel materials with customized characteristics and exceptional efficiency for a wide range of specialized applications such as tissue engineering techniques and drug delivery systems (DDSs), owing to its organizational nature and flexibility [105,106,107,108,109]. Regulated DDSs have piqued the attention of academic and industrial investigators since they were first authorized by FDA in 1990 [110]. As opposed to conventional medicine, medication systems can increase the bioavailability of the drug, maintain an adequate concentration of the drug, and reduce adverse reactions. PEGylated, Liposomes medication complex, systems based on PLGA, and protein-based mechanisms are all FDA-approved DDSs [111]. Because of their outstanding biocompatibility and pharmacokinetics, naturally existing polymer-based DDSs have recently gained a lot of publicity. Because of their uncommon combination of strong mechanical characteristics and manageable biodegradation ability, aqueous-based purification/processing, and medicine stabilization effect, SF-based substances are great options for the distribution of bioactive molecules among different groups of biopolymers. Therapeutic substances are generally maintained shipped to target locations, and distributed in a managed manner after being incorporated into the SF network [112].

Substances-based SF has been extensively studied for the delivery of antitumor substances that is the most significant title in biomedicine science, due to its drug stabilization potential. Poorly soluble in water chemotherapeutic agents can be stabilized, their bioactivity maintained after release, and therapy results improved thanks to the water-insoluble relationship with the fibroin network of β-sheet crystallites. Intratumoral and injectable administration have also been made possible with SF-based devices [113] because of the different formats that are available. For localized primary breast cancer treatment, Seib et al. used hydrogel of a SF-based to deliver doxorubicin (DOX) [114]. After sonication, aqueous SF fluids are combined with DOX and self-assembled into the thixotropic hydrogel. The packed DOX controllably discharged from the SF hydrogel after being infused locally into rats bearing breast cancer and it showed excellent tumor regression and decreased metastatic propagation. To induce the creation of nanofibers, Wu et al. prepared a DOX-loaded SF hydrogel and put it into a concentration–dilution cycle [115]. This device released DOX in a pH-responsive and concentration-dependent fashion, indicating that it may be a valuable method for regulating antitumor behavior. Phototherapy, over other chemotherapy and antitumor medications, is thought to be an effective therapeutic tool for removing tumors. Their photothermal effect and upconversion luminescence imaging output should be mixed; hydrogels based on SF have filled nanographene oxide compounds and lanthanide-doped special earth nanoparticles [116]. The theranostic hydrogels based on fibroin significantly decreased the measurement of treated tumors when exposed to NIR laser light. SF-based medicine carriers in the shape of nanomaterials are rapidly established and looked into for their possible application in intravenous, tumor care with systemic drug delivery, in contrast to hydrogel systems for cancer treatment on a local basis [117]. Furthermore, the capacity of anticancer drugs discharged from nanosized cargo to attack tumors will be advantageous for improved retention and permeability. Qu et al. described cisplatin-entrapped SF nanoparticles that were made by electrospraying [118]. Cisplatin may be distributed over 15 days thanks to its tight metal–ligand coordinate bonds with the SF matrix, and it had easy intracellular penetration and enhanced protective action on a lung tumor cell model. Tian et al. have created SF-based nanoparticles with DOX attached with the ability to target tumors magnetically [85]. The combined SF and superparamagnetic Fe_3_O_4_ nanoparticle method were rendered using the one-pot salting-out technique. DOX was delivered using external magnetic guides, resulting in good anticancer efficacy. MCF-7/ADR is a multidrug-resistant cancer cell line tumor, with a 30-day level of survival of up to 100 percent.

Nanoparticles from natural polymers, such as SF, stand out as ideal drug delivery systems due to their flexible nanostructures, biocompatibility compatibility, and custom degradation; however, the natural variability of polymers in the structure and release of drugs may limit their performance in selected conditions [117].

Larger particles are associated with the gradual release of the drug as the synthetic drug emanates from the nanoparticle area; in contrast, smaller particles indicate a faster release as the drug is closer to the nanoparticle surface [29]. In addition, small particles spread through tissues more easily than larger particles, leading to wider drug proliferation. Standing is also an important factor in the way nanoparticles behave. One study showed that circular nanoparticles had a much more efficient absorption than rod-shaped nanoparticles [23,119]. Chemical properties such as hydrophobicity and particle charge can determine the end of a target cell. Hydrophobic particles have a high potential for phagocytes, leading to targeted cell death [29].

Although the behavior of therapeutic nanoparticles is clearly multifactorial and sound conclusions cannot be drawn from in vitro therapeutic behavior, new research methods can be clarified. SF nanoparticles should be tested as a novel, in vivo system for drug delivery to wounds.

Investigators are particularly interested in using SF-based biomaterials to distribute natural possible anticancer compounds such as curcumin, in contrast to chemotherapy drugs such as DOX, paclitaxel, and cisplatin [69,120,121]. Apart from using hydrogels and nanoparticles as DDSs, there has been a lot of work put into using other SF-based biomaterials, such as medication distribution platforms. Coatings dependent on SF, for example, have been investigated as possible DDSs for tiny molecule medicines and biological agents. Bayraktar et al. coated theophylline tablets directly with a coating formulation for SF [122]. Biomedical uses include the distribution of biological particles such as proteins cell proliferation, genes, and peptides in contrast to the delivery of minute particles of drugs. As a result of its attributes, especially its tunable insolubility and very mild processing, SF may be a great member for immobilizing as well as the preservation of biological molecules. Li et al. detailed the beneficial stabilization SF biomaterials’ effect and process on biologics, such as enzymes, antibiotics, vaccines, and plasma molecules are only a few examples [94].

### 5.3. Controlled Drug Release

The goal of managed-release drug delivery systems is to release the embedded API in predetermined quantities over a predetermined period. Sustained-release rate is one use of such devices, which allows for the preservation of therapeutic drug doses in the bloodstream or at the action site for a prolonged period, which is important for the treatment of serious illnesses. PEG and PLGA are examples of the synthetic polymers used in the majority of presently available controlled-release formulations because they have attractive pharmacodynamic and pharmacokinetic characteristics [123]. Various functionalized SF for controlled or redirected medicines have been used (Table 4).

While the FDA has authorized PLGA as a safe ingredient in pharmaceuticals, processing conditions can limit its use in several controlled-release formulations. As a result, organic polymers such as SF, which provide kinetics of continuous release that can be tweaked and stabilization APIs that have been loaded, have currently attracted more interest for use in managed drug delivery systems. The capacity of SF to undergo various structural changes only at a molecular scale is one of its distinguishing characteristics. The increase in the ratio of alpha-helix to βeta-sheet material is the most studied structural transition in SF. The percentage of β-sheet formation, for example, influences the SF films’ permeability and release kinetics [7]. Hines and Kaplan previously explored the process of controlled release from science fiction films using numerous models [128]. The release of SF nanomaterials and microparticles under controlled conditions has been widely studied over the last decade. Song et al. [69] displayed curcumin release from SF nanoparticles is pH-controlled for up to 20 days to monitor SF molecules features, with lower pH facilitating the release. SF can be handled in aquatic conditions and crosslinked utilizing a variety of ways as a biopolymer. As a result, the SF solution has been utilized to cover a wide range of pharmaceutical formulations in single and multilayer coatings. The adenosine escape from SF-embedded powder reservoirs was computed as a characteristic of the reservoir coated layer, according to Pritchard et al. [129].

### 5.4. Gene Therapy

Bioengineered silk-elastin-like polymers (SELPs) have been commonly utilized as vectors for the transmission of plasmid DNA or adenoviral agents in modern genetic engineering scientific studies. SELPs’ chemical structure is made up of silk-like (Gly–Ala–Gly–Ala–Gly–Ser) and elastin-like (Gly–Val–Gly–Val–Pro) blocks that are tandemly replicated. Since they mix mechanical and thermal energy resilience of SF semi-crystalline frames with the solubility in water and durability of blocks of elastomeric elastin, silk-elastin-like polymers have been proposed. Furthermore, the ability to manipulate the monomer frames at the genomic level, as well as adenoviral viability is the unique feature of the substance that makes it ideal for gene therapy. Li et al. [130], used SELP hydrogels to distribute plasmid DNAs of various molecular weights and adeno-associated viral vector models. The DNA’s molecular mass and conformation, as well as the geometry of the hydrogel, regulated the drug release of encapsulated plasmid DNAs, and there was no substantial loss of bioactivity after 28 days. In a breast cancer-bearing mouse model, SELP hydrogel was used to deliver Renilla luciferase plasmid in vivo which resulted in a substantial improvement in luciferase gene expression and the preservation of transfection potency, which was the better option inside the tumor. Hatefi et al. [131] looked into adenoviral delivery using SELPs in vitro and in vivo. The stabilization virus interaction with silk or elastin units was due to the sustained release in vitro of imprisoned adenoviruses with the retained operation of infection after 28 days. Green fluorescent expression (GFP) was extended even 15 days after using virus-treated SELP samples after tumor treatments in vivo in xenograft murine systems, while the GFP term was reduced 11 days later with just the virus infusion, making this polymer helpful for localized adenovirus-mediated cancer therapy delivery. In the next, in a head and neck cancer xenograft model in mice, Ghandehari et al. [132] studied antitumors and the efficacy of an adenovirus-encoded SELP hydrogel. The SELP hydrogel with adenovirus was marginally more effective than the adenovirus/ganciclovir alone after 14 days of monitoring. The significant quantities of recent studies on the subject of utility, alteration, and construction of various SELPs have yielded promising results. Huang et al. studied the SELP-based patterned relationship of biomaterials of for the creation of different stimuli-responsive structures, as well as their present and future applications [133].

**Figure 3:**
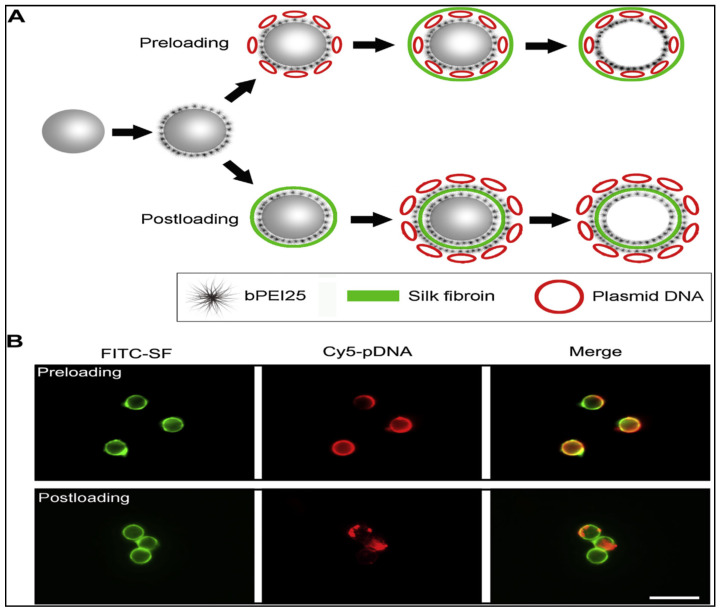
SF microcapsules of plasmid DNA are packaged. (**A**) A diagram depicting the initialization of pDNA before and after it has been loaded. Preloading: pDNA was adsorbed onto bPEI25 functionalized PS particles; SF was mounted on the pDNA-coated particles using LbL; the SF was stabilised, and the core was removed. PS particles were coated with bPEI25 after loading; SF was assembled LbL onto bPEI25-coated PS particles; the center was removed after pDNA was adsorbed onto the bPEI25eSF casing after another bPEI25 coating. (**B**) Photos obtained with a fluorescence microscope of 4 mm SF microcapsules that had been pre- or post-loaded with pDNA. FITC (**green**) was used to mark SF, and Cy5 was used to label pDNA (**red**). 10 mm scale bar. Reprinted with permission from reference [97].

Pre- and post-loading is used to integrate pDNA into SF microcapsules to find the best delivery mechanism in terms of the encapsulation of drugs, toxicity of cells, discharge of drugs, and the transfection performance. Prior loading was performed by electrostatically before SF deposition, adsorbing pDNA onto bPEI25-coated PS particles, resulting in nucleic acid/bPEI25 complexes entangled within the microspheres, with the SF multi-layer shell serving as both a defensive and a diffusion impediment (Figure 3A,B). In the post-loading process, the cargo is loaded onto or into pre-fabricated capsules by adsorption and/or diffusion through the capsule casing (Figure 3A,B) [134]. Several studies [135,136,137] have successfully employed these techniques and the loading process and shell thickness is used to customize drug release characteristics [138].

**Figure 4:**
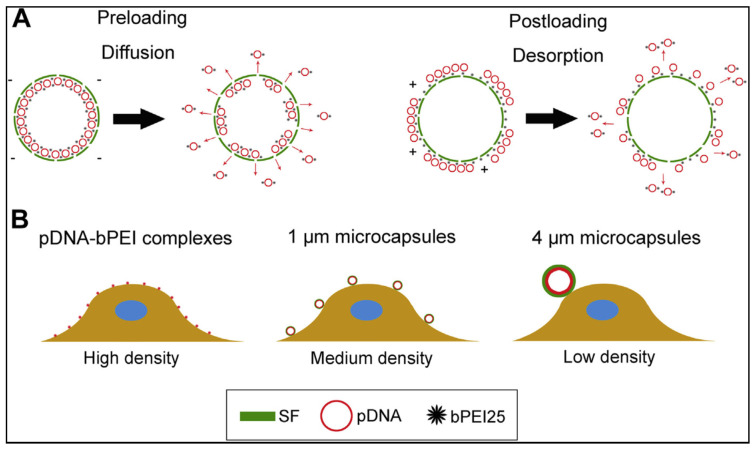
The results of various loading methods and measures of pDNA loaded SF micro capsules on release activity and cell capsule interactions are depicted in this diagram. (**A**) Preloading: pDNAebPEI complexes are released from SF capsules through diffusion. After charging, desorption from its membrane of SF capsules releases complexes. (**B**) For the same total amount of pDNA, varying sizes of gene carriers have various transmission densities on the cell surface, affecting cell viability and transfection. Reprinted with permission from reference [97].

Sustained pDNA release was observed in both classes, as measured by the decrease in Cy5-pDNA fluorescence associated with microcapsules, which was substantially in the presence of Protease XIV, the concentration is greater than in pure PBS. In the case of preloaded microcapsules, discharge is thought to occur as a result of absorption and desorption, while pDNA release after post-loading desorption is believed to be the cause (Figure 4A). According to Lu et al., the first hydrophilic blocks are damaged through protease, blocks with high crystallinity are left behind, eventually move into the solution as particles [139]. Since shell porosity, pDNA release, and desorption all increased as SF depleted, the protease was significantly both preloaded and post-loaded microcapsule improved. We also discovered that after pre- and post-loading, the transfection performance of 1 mm microcapsules was slightly higher than that of 4 mm microcapsules. We believe that the density of microcapsules on the cell surface causes this effect, which leads to a greater likelihood of encounters and better touch with the surface of the cell as capsule size is reduced (Figure 4B). Although further research is needed, this result may be another influence on capsule size in cytotoxicity. Even though it was less distinct than its effect on transfection efficiency, it was a significant parameter for balancing cytotoxicity and transfection quality.

When contrasted to pDNA/bPEI25 complexes, SF microcapsules filled with plasmid DNA transduced NIH/3T3 fibroblasts successfully, eliminating cytotoxic effects. The findings indicate that SF microcapsules have the potential to be a powerful carrier for regulated, localized gene delivery.

### 5.5. Wound Healing

Hemostasis, inflammation, replication, and remodeling are all part of the wound healing process, which is a fluid and dynamic process. Weak mechanical efficiency, high expense, collagen inconstancy, poor processability, restricted supply of elastins, soft silicons, and polyurethanes being non-biodegradable are all disadvantages of the currently used wound dressing products. Because of its availability, inherent biodegradation ability, biocompatibility, mechanical robustness, signaling molecules stability ability, high synthesis of water and oxygen, and poor immunogenicity, SF may be considered an outstanding wound healing material (Table 5). SF facilitates regeneration by more complex pathways and signaling pathways, which have been briefly summarised by Farokhi et al. in addition to the above-mentioned properties [140].

Injury covering matrices are normally filled with growth factors to facilitate epithelization, in contrast to antimicrobial compounds to avoid the growth of bacteria. Schneider et al. tested the tissue repair ability of epidermal growth factor-containing electrospun (EGF) silk mats on human skin-equivalent models [150]. Gil et al. studied the impact of various silk fiber content layouts and in related research, the effects of the drug loading approach on wound healing in vivo were studied and aimed at evaluating the feasibility of EGF/silver sulfadiazine/SF systems on wound covering applications [151]. Woong et al. reportedly immobilized a wound-healing antimicrobial peptide, Cys-KR12, onto an SF nanofiber layer [152]. It has been reported that insulin-loaded SF microparticles embedded within SF sponges are used to treat chronic cutaneous wounds [153]. Keratinocytes and endothelial cells can experience enhanced multiplication and differentiation as a result of insulin’s stimulatory impact. The use of SF-calcium alginate-carboxymethyl cellulose hydrogel, an SF-based blending composite, for the treatment of burn wounds was stated by Kim et al. [154]. Vasconcelos et al. also created a blending hydrogel device that merged elastin’s durability, elasticity, and silk’s tunable biodegradation and high mechanical strength made it ideal for use as a wound dressing medium [155]. Zhang et al. carried out a unique translational study in which clinically focused and extensive preclinical experiments were carried out on full-thickness skin defect models in rabbits and porcines, as well as randomized controlled clinical trials on human subjects [156].

**Figure 5:**
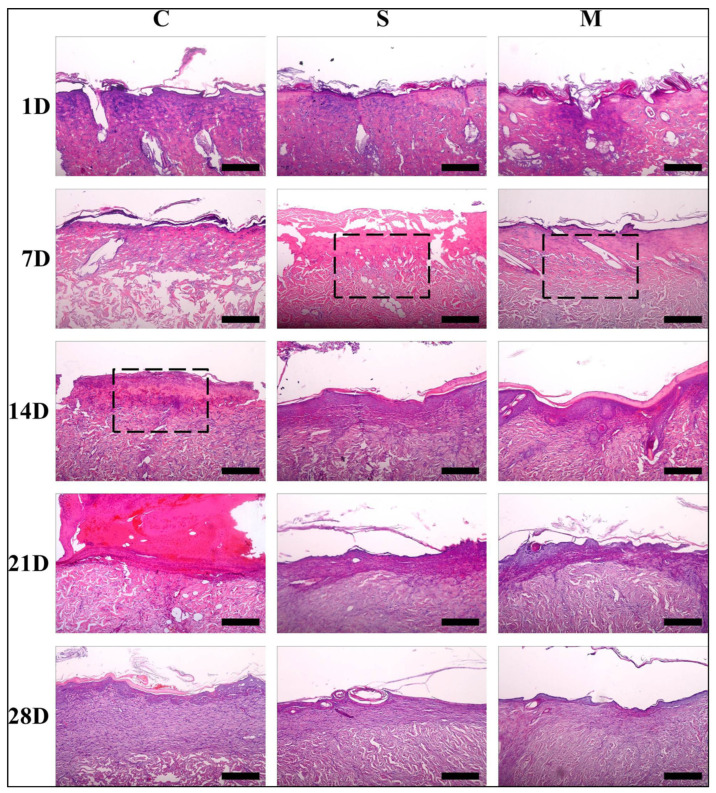
Burn wound tissues stained with hematoxylin and eosin (C: surgical gauze, S: SF nanomatrix, and M: Medifoam^®^, scale: 100 µm). Reprinted with permission from reference [157].

According to Ju et al., [157] they used a modified electrospun device combined with a mimetics (i.e., sodium chloride crystal) dispensing apparatus to fabricate SF nano matrix with total density and wide holes, and we checked the burn tissue repair effect in rats using a deep second-degree burned mouse model. Histological findings were used to examine the wound recovery mechanism and an RT-PCR assay was used to establish the healing process in contrast to a freely available market dressing (i.e., polyurethane foam and Medifoam^®^). They looked at histological changes in damaged skin to see if the SF nano matrix treatment affected wound healing (Figure 5). On day one, the presence of a blister and edema indicated a second-degree burn without any tissue injury to the underlying fascia and muscle tissue.

**Figure 6:**
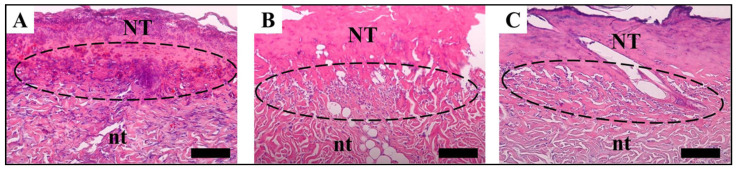
Photos of burn wound tissues stained with H&E at 7, 14, and 21 days. (**A**) Hospital gauze (14 days), (**B**) SF nanomatrix (7 days), and (**C**) Medifoam^®^ (7 days). (Scale: 50 µm, NT: necrosis tissue, nt: natural tissue, circle: Keratinocytes). Reprinted with permission from reference [157].

**Figure 7:**
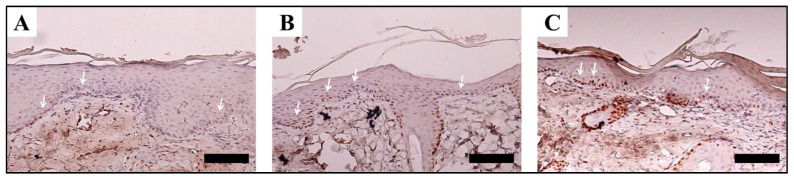
At 7 days, PCNA expression was observed in the tissue covering the infected region. Medical gauze (**A**), SF nanomatrix (**B**), and Medifoam^®^ (**C**) (scale: 50 µm). Reprinted with permission from reference [157].

As contrasted to the medical gauze-treated community, the collagen array in the SF nanomatrix and Medifoam^®^-treated groups was much thicker and far more constant [157]. Scar elimination, collagen and epithelialization, and PCNA expression are all factors to consider both confirmed that the SF nanomatrix could speed up the recovery of severe burns in rats (Figure 5, Figure 6 and Figure 7).

The mechanism of re-epithelialization started seven days after operation in both classes. The nano matrix party at SF, in particular, demonstrated quicker cell proliferation in the burned area (S), whereas clots of fibrin existed on the wounded area’s surface in the party that was treated with surgical gauze (C). Groups treated with SF nanomatrix and Medifoam^®^ (S and M) demonstrated quicker processes of re-epithelialization than the surgical gauze-treated community over the entire healing cycle (C) (Figure 6).

**Figure 8:**
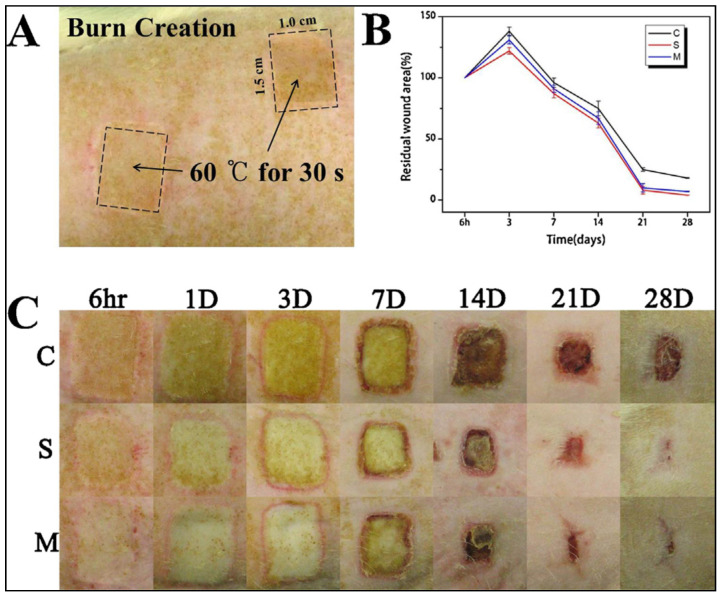
(**A**) Burn wound area on rat skin right after the creation. (**B**) Residual wound area change with healing time (28 days). (**C**) Gross findings of wound area treated with different wound dressing materials (C: medical gauze, S: SF nanomatrix, and M: Medifoam^®^) (scale: 50 µm). Reprinted with permission from reference [157].

The wounds managed with the SF nano matrix had morphogenesis and histology compared to usual skin after 14 days, and the wound region had regenerated without edema or granulation tissue. The medical gauze party, on the other hand, demonstrated extreme neutrophil and lymphocyte infiltration. On day 28, collagen accumulation at the injury site is seen in Figure 8.

Within 14 days after treatment, wound healing improved as the wounds were treated with SF nanomatrix skin regeneration and cell aggregation relative to injury healing with medical gauze, according to the wound size measurements (Figure 8). SF nanomatrix’s rapid wound healing may be due to a variety of factors. Human fibroblasts and keratinocytes have been shown to proliferate when exposed to SF [158], as well as collagen deposition [159].

## 6. Conclusions

Silk is a flexible biomaterial with several promises in terms of gene and drug delivery. SF has been used to make SF films, hydrogels, microparticles, and nanoparticles, among other drug delivery methods, employing a variety of manufacturing procedures. Every one of these SF-based frameworks has represented promise in a range of biomedical applications. Curcumin, doxorubicin, and ibuprofen, as well as pDNA, have all been delivered to different types of cells utilizing SF micro- and nanoparticles in a time and site-specific way. Drugs such as dextran and epirubicin, as well as biological agents such as IgG and HIV inhibitor 5P12-RANTES, have been controlled released using SF films. Furthermore, they have been used to keep biomedical agents such as horseradish peroxidase (HRP), oxidase of glucose, vaccines, and monoclonal antibodies fresh for longer. SF has also been used to extend the release and biological role of biomolecules including insulin and BMP-2. SF has been loaded with specific biological elements such as the RGD sequence, folate, and Her2 for tissue-specific drug delivery. SF has been used to cover the surfaces of polymer micro materials and liposomes to alter their release kinetics or improve cell adhesion, in comparison to drug carriers that depend on it. Another area that requires further study is changing the SF’s physicochemical and mechanical properties by mixing it with other inorganic fillers to create engineered SF-based biomaterials. Furthermore, due to their tunable properties, SF-based drug delivery systems open up new possibilities for drug delivery, gene therapy, and wound healing.

## Figures and Tables

**Table 1:** Preparation method of silk fibroin (SF) micro- and nanoparticles.

S. N.	Preparation Technique	Advantages	Disadvantages	Particle Size	Ref.
01	Freeze drying	Porous particles	Temperature dependent	490–940 nm	[14]
02	Self-assembly	Simple and safe technique Avoidance of toxic solvents	Prevent intermolecular	100–200 nm	[30]
03	PVA Blending method	Time and energy efficient No use of organic solvent	PVA filtrate	300–400 nm	[81]
04	Salting out	Economical technique The drug can be encapsulated at the time of particle formation	Salting out agents filtrate	500 nm–2 µm	[84]
05	Microfluidic methods	Rapid technique Mild operation procedures Controlled particle size	Complex process	150–300 nm	[86]
06	Emulsification	Controllable particle size Low-cost method	Residual surfactant	170 nm	[88]
07	Desolvation	Simple and quick method Small particle size Reproduceable technique	Easy to amassed; low drug load	35–170 nm	[89]
08	Electrospraying	High-purity particles Very good monodispersity	Requires post handling to make insolubility of SF	59–80 nm	[90]

**Table 2:** SF-based drug delivery systems.

Form of Drug Delivery System	Linked API	Outcome	Ref.
SF sponges	Erythromycin	Sustained drug release and extended antimicrobial effects against *S. Aureus*	[3]
SF nanoparticles	Curcumin	Modified drug release pattern and increased cellular uptake	[68]
Modified the release profile	Ibuprofen	Increased adhesion and tunable drug release	[93]
SF films	Epirubicin	Controlled drug release	[94]
SF microspheres	Horseradish peroxidase (HRP)	Modified the release profile	[95]

**Table 3:** SF-based formulations for gene delivery.

Formulation	Gene	Cell line	Outcome	Ref.
Bioengineered silk films	pDNA (GFP)	Human HEK cells	Beneficial for refining together the transfection efficiency and cell viability	[96]
SF layer-by-layer assembled microcapsules	pDNA-Cy5	NIH/3T3 fibroblasts	Uniting low cyto-toxicity and high transfection effectiveness	[97]
Bioengineered silk–polylysine–ppTG1 nanoparticles	pDNA	Human HEK and MDA-MB-435 cells	Improves transfection efficiency	[98]
Magnetic-SF/polyethyleneimine core-shell nanoparticles	c-Myc12 antisense ODNs	MDA-MB-231 cells	Meaningfully advanced inhibition effect	[99]
3D porous scaffold	Adenovirus Ad-BMP7	Human BMSCs	Extended term compatibility for growth factor	[100]

Abbreviations: GFP = green fluorescent protein, ODN = oligodeoxynucleotides, BMP = bone morphogenic protein.

**Table 4:** Various functionalized SF for controlled or redirected medicines.

SF Biomaterial	Applications	References
SF nanofibers	Drug delivery system	[115,124]
SF spheres	Controlled drug delivery	[81,125]
SF matrices	Drug delivery and controlled release	[126]
SF Nanoparticles	Drug delivery	[127]

**Table 5:** SF-based drug delivery systems for wound healing.

SF Biomaterial	Applications	References
SF solutions	Skin wound repair	[40,141]
SF hydrogels	Wound healing drug delivery	[142,143,144]
SF Nanoparticles	Drug delivery	[145,146,147]
SF biosensors	Wound monitoring	[148,149]

## Data Availability

This article did not report any data.
